# Synchronous gastric and appendiceal mucinous adenocarcinomas: a rare case report

**DOI:** 10.3389/fonc.2026.1760066

**Published:** 2026-04-15

**Authors:** Guangchao Liu, Cheng Jiao, Sen Wang, Shuai Qi, Cuisha Zhang, Qiufang Xing, Wei Liu

**Affiliations:** Department of General Surgery, Bethune International Peace Hospital, Shijiazhuang, Hebei, China

**Keywords:** appendiceal mucinous adenocarcinoma, case report, gastric mucinous adenocarcinoma, microsatellite instability, pseudomyxoma peritonei

## Abstract

**Background:**

Gastric mucinous adenocarcinoma (GMA) and appendiceal mucinous adenocarcinoma (AMA) are rare neoplasms that frequently present at advanced stages characterized by peritoneal dissemination. The simultaneous occurrence of these two malignancies is exceedingly uncommon.

**Case presentation:**

A 66-year-old man presented with abdominal discomfort and was initially diagnosed with gastric adenocarcinoma based on endoscopic biopsy findings. Computed tomography imaging revealed the presence of ascites and peritoneal nodules. Following neoadjuvant chemoimmunotherapy comprising XELOX and sintilimab, diagnostic laparoscopy identified a concurrent AMA accompanied by pseudomyxoma peritonei (PMP). Subsequently, the patient underwent radical gastrectomy, appendectomy, and hyperthermic intraperitoneal chemotherapy (HIPEC). Histopathological examination confirmed the synchronous presence of GMA and AMA. Importantly, serial molecular analyses demonstrated a treatment-induced transition from mismatch repair-deficient (dMMR)/microsatellite instability-high (MSI-H) status to mismatch repair-proficient (pMMR) status.

**Conclusion:**

This rare case highlights the critical importance of comprehensive preoperative imaging and meticulous surgical evaluation in the diagnosis of uncommon synchronous malignancies. Furthermore, the observed alteration in molecular phenotype following chemoimmunotherapy suggests that such treatment modalities may influence tumor biology.

## 1 Introduction

Gastric mucinous adenocarcinoma (GMA) and appendiceal mucinous adenocarcinoma (AMA) are rare and aggressive subtypes of gastric and appendiceal cancers, respectively. GMA is characterized by abundant extracellular mucin production and frequently presents at an advanced stage with peritoneal dissemination (1). AMA is notably associated with the development of pseudomyxoma peritonei (PMP) due to excessive mucin secretion (2). The simultaneous occurrence of these two malignancies is exceedingly uncommon, with no documented cases reported in the literature over the past decade. This concurrence poses distinct diagnostic and therapeutic challenges.

A 66-year-old man presented with abdominal discomfort. Endoscopic biopsy confirmed the diagnosis of gastric adenocarcinoma. Computed tomography (CT) imaging revealed ascites and peritoneal nodules indicative of peritoneal metastases. Neoadjuvant chemoimmunotherapy was administered to facilitate subsequent surgical intervention. The primary appendiceal lesion was not identified on initial imaging, likely owing to its atypical presentation and subtle radiographic features. Subsequent diagnostic laparoscopy uncovered extensive intra-abdominal mucinous deposits and a perforated appendix, thereby establishing the presence of synchronous AMA. The patient underwent laparoscopy-assisted distal gastrectomy with D2 lymphadenectomy, appendectomy with partial cecal resection, and complete cytoreductive surgery. Histopathological examination confirmed the diagnosis of synchronous primary GMA and AMA. These findings underscore the critical importance of thorough preoperative imaging reassessment and meticulous intraoperative exploration to ensure accurate diagnosis and staging in cases involving rare synchronous malignancies.

## 2 Case description

**Figure 1:**
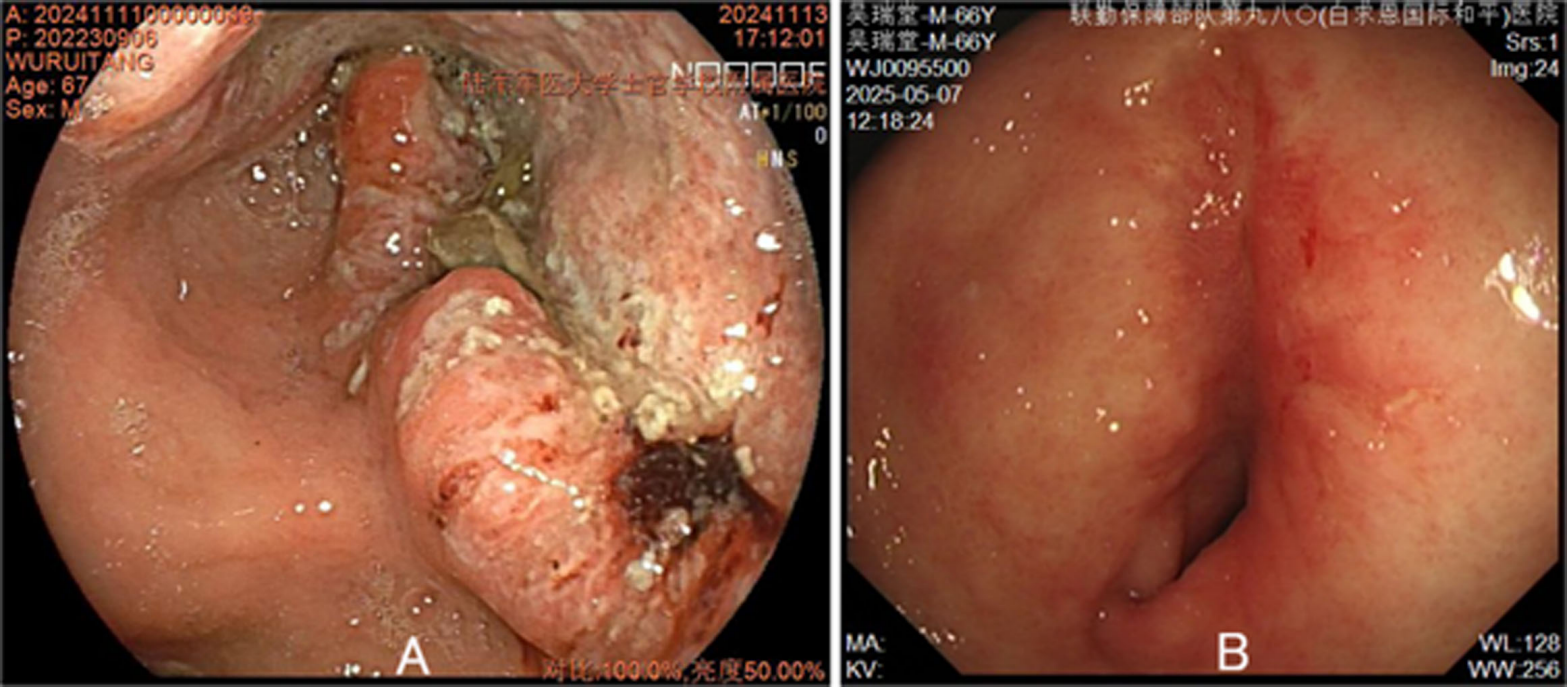
Endoscopic appearance of the gastric antral mass before **(A)** and after **(B)** conversion therapy.

A 66-year-old man presented on 14 November 2024 with six months of intermittent dull epigastric pain and postprandial fullness. Pre-admission gastroscopy had demonstrated a large cauliflower-shaped antral mass causing luminal obstruction (**Figure 1A**); biopsy confirmed adenocarcinoma (**Supplementary Figure S3**). He had no significant medical history.

**Figure 2:**
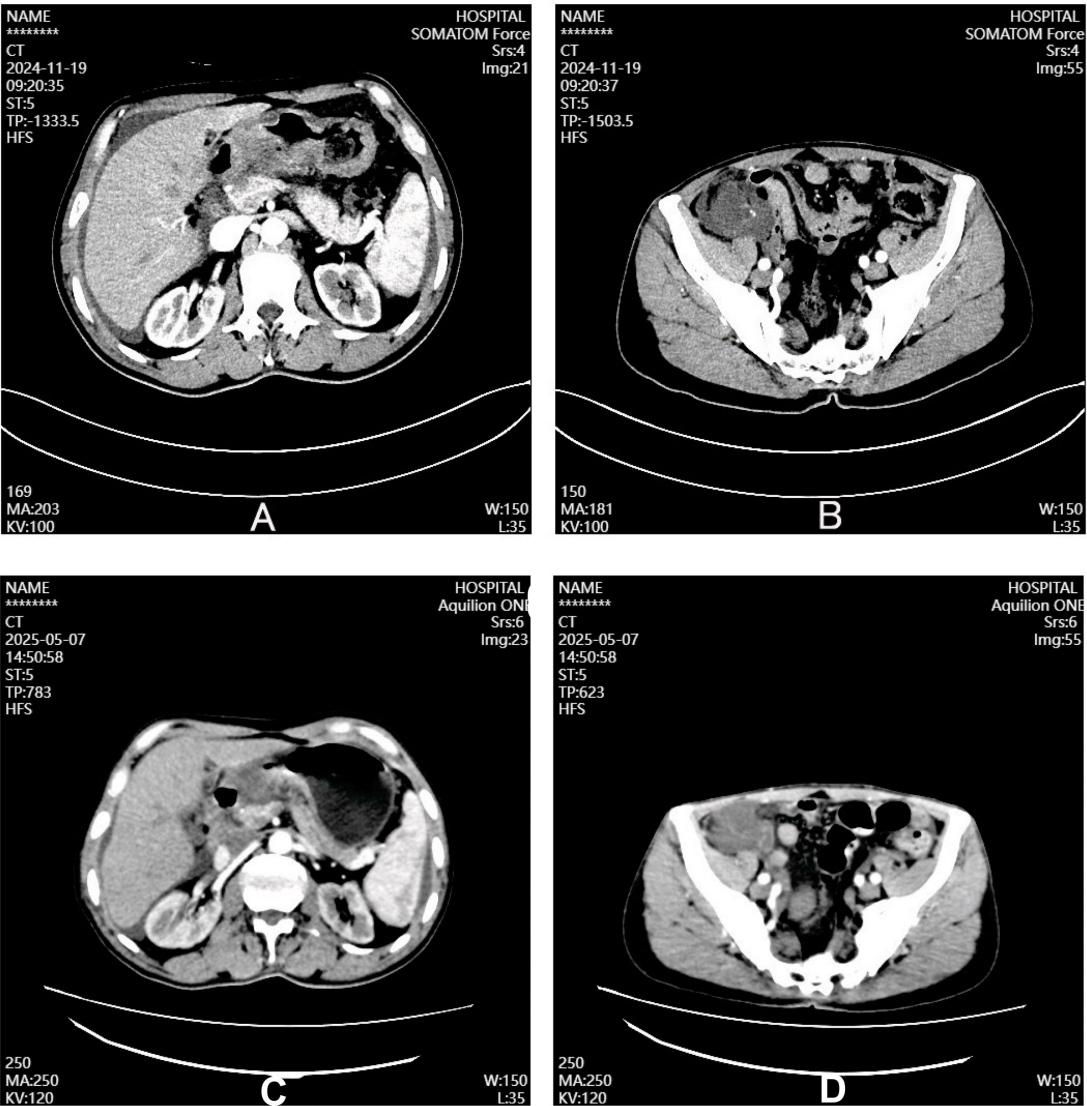
Contrast-enhanced CT findings before **(A, B)** and after **(C, D)** neoadjuvant chemoimmunotherapy. Baseline imaging demonstrated marked gastric wall thickening with luminal narrowing **(A)**, omental infiltration, moderate ascites, and a cystic lesion with irregular wall thickening in the right iliac fossa **(B)**. Following six cycles of XELOX plus sintilimab, significant regression of gastric wall thickening was observed with improved luminal patency **(C)**; however, ascites persisted and the right lower quadrant cystic lesion remained essentially unchanged **(D)**.

On admission, his body mass index (BMI) was 19 kg/m² and Eastern Cooperative Oncology Group (ECOG) performance status 0. Physical examination revealed isolated upper abdominal tenderness without guarding or palpable masses. Laboratory tests showed elevated tumor markers: carcinoembryonic antigen (CEA) 7.95 ng/ml and carbohydrate antigen 724 (CA724) 51.1 U/ml; CA125 was within normal limits (25.87 U/ml). Contrast-enhanced abdominal CT demonstrated malignant gastric wall thickening with omental infiltration, ascites, and a cystic lesion with irregular wall thickening in the right lower quadrant near the pelvic inlet (**Figures 2A, B**). Initial immunohistochemistry of the gastric biopsy revealed mismatch repair protein deficiency (dMMR): MLH1(+), MSH2(-), MSH6(-), PMS2(+); HER-2 status was negative (0).

Following a multidisciplinary team (MDT) review in line with National Comprehensive Cancer Network (NCCN) guidelines, the patient underwent six cycles of XELOX (capecitabine 1500 mg twice daily on days 1-14; oxaliplatin 180 mg on day 1) plus sintilimab (200 mg on day 1) every 3 weeks as conversion therapy. Post-treatment restaging CT showed significant gastric tumor regression with reduced wall thickening and improved luminal patency; however, ascites persisted and the right iliac fossa cystic lesion remained essentially unchanged (**Figures 2C, D**). The right lower quadrant cystic lesion, unchanged from baseline, was preoperatively interpreted as a probable ovarian or colonic cyst; appendiceal neoplasm was not initially suspected. Repeat gastroscopy confirmed marked tumor reduction with no macroscopic residual disease (**Figure 1B**).

**Figure 3:**
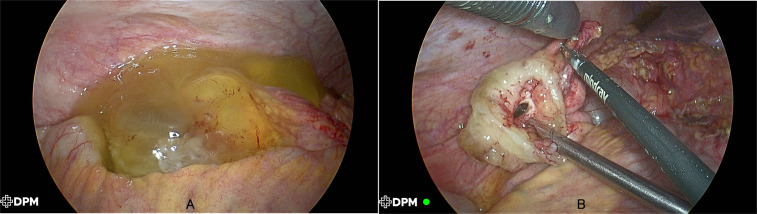
Laparoscopic findings: perforated appendix, and mucinous deposits.

On 7 May 2025, the patient was readmitted for surgical exploration. Preoperative multidisciplinary planning included consent for possible HIPEC should peritoneal disease be confirmed. Diagnostic laparoscopy revealed approximately 200 ml of pale yellow ascites and extensive mucinous deposits coating the liver, small bowel, colonic serosa, and pelvic organs. The gastric antrum appeared thickened, and the appendix was perforated with adherent mucin, corresponding to the previously observed cystic lesion (**Figure 3**). Intraoperative findings were suggestive of a perforated mucinous appendiceal neoplasm. Frozen-section analysis was not utilized, as definitive characterization of mucinous neoplasms generally benefits from permanent section evaluation with ancillary immunohistochemical studies. In light of the operative duration already incurred and the patient’s condition following extensive cytoreduction, right hemicolectomy was deferred to a subsequent procedure following nutritional recovery. These findings prompted immediate definitive surgery, and the patient underwent laparoscopy-assisted radical distal gastrectomy, appendectomy with partial caecectomy, cytoreductive surgery, and hyperthermic intraperitoneal chemotherapy (HIPEC) in the same session. Complete macroscopic cytoreduction was achieved (CC-0 score), with no visible residual mucinous deposits or tumor nodules remaining at the conclusion of the procedure.

Final histopathology confirmed synchronous primary malignancies: GMA (pT4aN0, 0/31 lymph nodes, tumor regression grade (TRG) 1, mucin component ≥50%, negative margins) and AMA (full-thickness invasion with PMP features). The gastric lesion (3.0×3.0×1.5 cm) on the lesser curvature showed acellular mucin pools with focal adenocarcinoma clusters, serosal invasion, and no lymphovascular or perineural invasion. The appendiceal lesion (3.0 cm) demonstrated villous serrated adenoma with high-grade dysplasia and acellular mucin infiltration into the muscularis propria and serosa. Post-treatment microsatellite instability (MSI) testing revealed proficient mismatch repair (pMMR): MLH1(+), MSH2(+), MSH6(+), PMS2(+), indicating conversion from baseline dMMR (MSI-high). Immunohistochemistry confirmed distinct primaries: gastric (MUC5AC-, P53-, mutant, Ki-67 60-70%) and appendiceal (MUC5AC+, P53 wild-type expression, Ki-67 8-10%) (**Supplementary Figures S1**, **S2**).

Postoperative management included intravenous nutrition, antibiotics, and proton-pump inhibition. Oral intake was gradually resumed, and the patient was discharged after one month. Right hemicolectomy was deferred due to suboptimal nutritional status and recent major gastrectomy. Adjuvant therapy comprised eight cycles of single-agent capecitabine, selected for its activity against both mucinous primaries and the patient’s limited tolerance for platinum-based regimens following extensive surgery and prior oxaliplatin exposure. Sintilimab maintenance was continued for 12 months given the marked neoadjuvant response. At six-month follow-up, he remained disease-free with stable nutritional status. The patient reported that the initial diagnosis caused significant anxiety, though detailed explanation of the treatment plan helped him proceed. During neoadjuvant therapy, he experienced mild fatigue and nausea but continued treatment without interruption, noting improved postprandial symptoms after the second cycle. He found the postoperative recovery demanding yet understood the rationale for staging the surgeries. At six months, he had returned to normal activities and was satisfied with the outcome (**Table 1**).

**Table 1:** Timeline with relevant data.

Date	Event
2024-11-14	The patient presented with abdominal discomfort.
2024-11-21	Gastroscopy revealed a large antral mass; biopsy confirmed adenocarcinoma. Contrast-enhanced CT showed gastric wall thickening, omental infiltration, ascites, and a cystic lesion in the right iliac fossa.
2024-12-01	Multidisciplinary team (MDT) recommended conversion therapy with six cycles of XELOX plus sintilimab.
2025-03-27	Completion of conversion therapy. Restaging CT demonstrated significant tumor regression but persistent ascites.
2025-05-07	Diagnostic laparoscopy revealed extensive mucinous deposits and a perforated appendix. The patient underwent laparoscopy-assisted radical distal gastrectomy (D2), appendectomy with partial caecectomy, cytoreduction (CC-0), and HIPEC.
2025-05-17	The patient was discharged after postoperative recovery. Adjuvant therapy with capecitabine and sintilimab maintenance therapy initiated.
2025-11-17	6-month follow-up showed the patient remains disease-free with stable nutritional status.

## 3 Discussion

GMA, defined by extracellular mucin exceeding 50% of tumor volume (3), accounts for 3-7% of gastric adenocarcinomas. This aggressive variant typically presents at advanced stage with peritoneal dissemination and lymphatic invasion, yielding substantially lower 5-year survival than non-mucinous counterparts (4). Molecular analyses indicate a predominance of HER2 negativity or low expression levels, a low frequency of CLDN18.2 positivity, and an increased prevalence of dMMR (5–7). Diagnostic precision is enhanced through the use of 68Ga-FAPI PET/CT imaging, which demonstrates superior performance compared to 18F-FDG PET/CT (8). From a therapeutic perspective, neoadjuvant chemotherapy yields a pathologic complete response (pCR) rate of 38% in GMA cases, significantly exceeding the 10% rate observed in diffuse-type gastric cancer; however, postoperative peritoneal recurrence remains a significant clinical challenge (3).

AMA accounts for approximately 20-40% of tumors arising in the appendix and primarily affects middle-aged to elderly individuals, with no observed sex predilection (9, 10). These neoplasms are characterized by excessive mucin production, frequently leading to PMP, a condition marked by the accumulation of mucinous material within the peritoneal cavity (2, 11). Clinically, AMA often presents with nonspecific symptoms that may mimic acute appendicitis or ovarian malignancies; thus, diagnosis depends on imaging findings such as mucoceles or the “onion-skin” appearance on CT, in conjunction with histopathological confirmation (12). Molecular studies have identified activation of the KRAS, GNAS, and MYC signaling pathways as critical drivers in mucinous adenocarcinomas, aiding in the classification of subtypes and the development of targeted therapies (2, 13). The current standard of care involves cytoreductive surgery combined with HIPEC, which has been associated with five-year survival rates reaching up to 80% in patients presenting with peritoneal metastases (14).

The diagnosis of synchronous primary cancers relies on demonstrating histological independence and non-metastatic nature. In our case, the gastric lesion exhibited acellular mucin pools with focal adenocarcinoma clusters and serosal invasion, while the appendiceal lesion showed villous serrated adenoma with high-grade dysplasia and mucin infiltration into the muscularis propria. The presence of a precursor lesion in the appendix and divergent differentiation patterns supported independent tumorigenesis rather than metastatic spread. Molecular profiling revealed distinct immunohistochemical markers: gastric (MUC5AC-, P53-mutant, Ki-67 60-70%) versus appendiceal (MUC5AC+, P53 wild-type, Ki-67 8-10%). While MUC5AC supported distinct immunophenotypes, we recognize its limited organ specificity. SATB2, had it been available, would have strengthened the diagnostic certainty; similarly, neoadjuvant therapy may have altered the appendiceal immunophenotype in the absence of pre-treatment tissue. However, the preserved villous serrated architecture, markedly divergent Ki-67 indices, and opposing MUC5AC and P53 expression patterns are unlikely to result from differential chemotherapy response and collectively favor synchronous primaries. Initial symptoms, gastroscopy, and imaging suggested two distinct tumors, confirmed by diagnostic laparoscopy showing extensive mucinous deposits and a perforated appendix indicative of AMA with PMP.

Accurate preoperative staging is critical for effective therapeutic planning in gastric cancer. Although CT and MRI provide reliable evaluation of tumor extent and metastatic spread, subtle lesions may be missed (15). In the present case, endoscopic biopsy established the diagnosis of gastric adenocarcinoma, while CT imaging revealed ascites and omental nodules indicative of peritoneal metastases. Consequently, conversion therapy was initiated. However, the atypical presentation of AMA led to its omission from the initial differential diagnosis, resulting in incomplete staging. A retrospective review of imaging identified a cystic-solid lesion with wall thickening in the right iliac fossa, partially obscured by adjacent bowel loops. Subsequent diagnostic laparoscopy confirmed appendiceal pathology and enabled definitive staging. These observations highlight the importance of thorough imaging reassessment and the role of diagnostic laparoscopy in patients with advanced gastric cancer undergoing conversion therapy to ensure accurate staging.

The mismatch repair (MMR) system plays a crucial role in preserving genomic stability by rectifying DNA base mismatches. Mutational or epigenetic inactivation of MMR genes, including MLH1, MSH2, MSH6, and PMS2, leads to MMR deficiency (dMMR) and subsequent high levels of microsatellite instability (MSI-H) (16). In gastrointestinal malignancies, MSI status serves as a pivotal biomarker for prognostication and guiding immunotherapeutic interventions. By definition, dMMR corresponds to MSI-H, whereas proficient MMR (pMMR) is associated with microsatellite stability (MSS) (17). Initial gastric biopsy findings indicated dMMR/MSI-H status (MLH1+/MSH2-/MSH6-/PMS2+), which informed the initiation of combined chemoimmunotherapy. However, postoperative immunohistochemical analysis revealed a shift to pMMR status (MLH1+/MSH2+/MSH6+/PMS2+), suggesting phenotypic plasticity potentially resulting from therapy-induced alterations in clonal composition or MMR protein expression. Given the pronounced spatial and temporal heterogeneity characteristic of gastric cancers (18), fluctuations in MMR status during treatment are plausible (19). Although the transition from dMMR to pMMR may theoretically reduce the effectiveness of immunotherapy, the patient exhibited significant initial tumor regression. Notably, sintilimab has demonstrated efficacy even in populations with low PD-L1 expression. In light of the favorable clinical response, the multidisciplinary team decided to continue treatment with sintilimab in combination with capecitabine to consolidate therapeutic benefits.

The classification of appendiceal mucinous tumors has undergone ongoing refinement. According to the 2010 WHO classification, these tumors are categorized into three distinct groups: mucinous adenoma, low-grade appendiceal mucinous neoplasm (LAMN), and AMA (20). Management strategies differ among these categories; mucinous adenomas are typically treated with simple appendectomy, while patients diagnosed with LAMNs and AMAs have demonstrated improved survival outcomes following right hemicolectomy (20). Pathologic analysis confirmed AMA with PMP features in this case (21). Right hemicolectomy was postponed owing to malnutrition following gastrectomy and an extended recovery period. HIPEC was performed intraoperatively to manage peritoneal involvement. The patient subsequently completed adjuvant chemoimmunotherapy, and ongoing surveillance is being maintained.

## 4 Conclusion

Synchronous GMA and AMA has not been reported in the past decade. We describe a 66-year-old man diagnosed with gastric adenocarcinoma on initial gastroscopy, whose staging CT suggested omental metastasis. After conversion therapy, diagnostic laparoscopy surprisingly revealed concurrent AMA with PMP, leading to radical gastrectomy and appendectomy that confirmed both primary tumors. This case illuminates three imperatives: (1) comprehensive preoperative re-evaluation and heightened suspicion for synchronous rare neoplasms; (2) therapeutic strategies tailored to tumor biology and patient status; and (3) the capacity of chemoimmunotherapy to effect phenotypic shifts, evidenced by the dMMR-to-pMMR conversion. These observations warrant refinement of staging protocols and personalized, molecularly-driven management algorithms for uncommon presentations.

## Data Availability

The datasets presented in this study can be found in online repositories. The names of the repository/repositories and accession number(s) can be found in the article/**Supplementary Material**.
