# Supplementary material for: Synchronous gastric and appendiceal mucinous adenocarcinomas: a rare case report
Source: Front Oncol. 2026 Apr 15;16:1760066. doi: 10.3389/fonc.2026.1760066 (PMC13124552; doi:10.3389/fonc.2026.1760066)
Supplement: Supplementary file 1 [file DataSheet1.docx]

**Figure S1. Histopathological and immunohistochemical features of gastric mucinous adenocarcinoma**

| 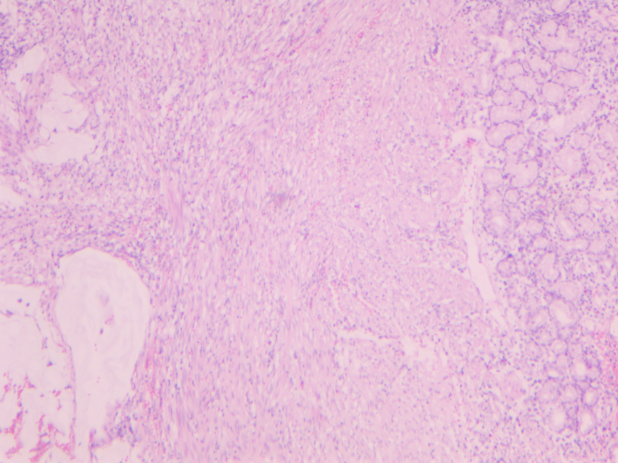 | 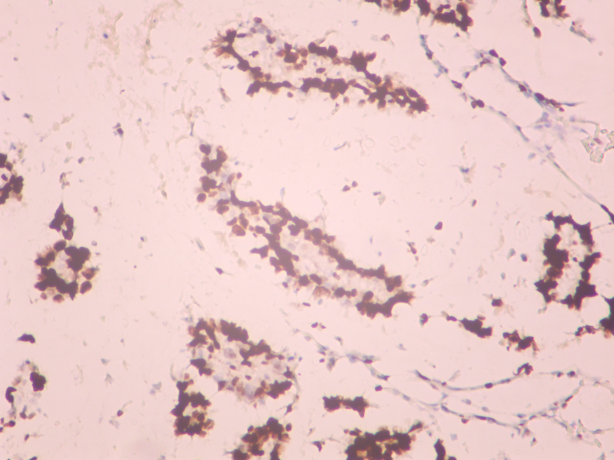 |
| --- | --- |
| H&E staining | KI-67: 60-70% |
| 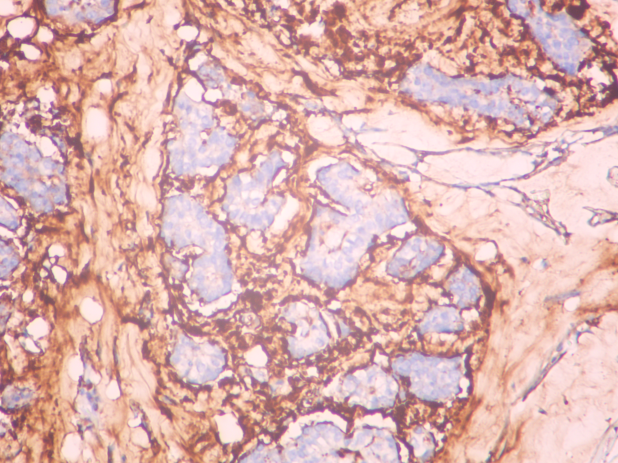 | 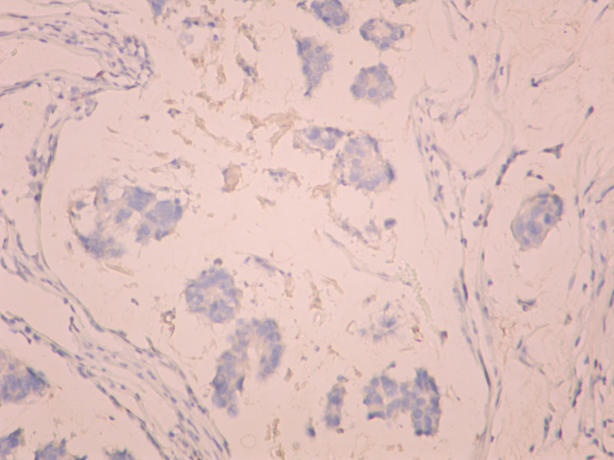 |
| MUC5AC: negative | P53: -, mutant |

**Figure S2. Histopathological and immunohistochemical findings in appendiceal adenocarcinoma tissue**

| 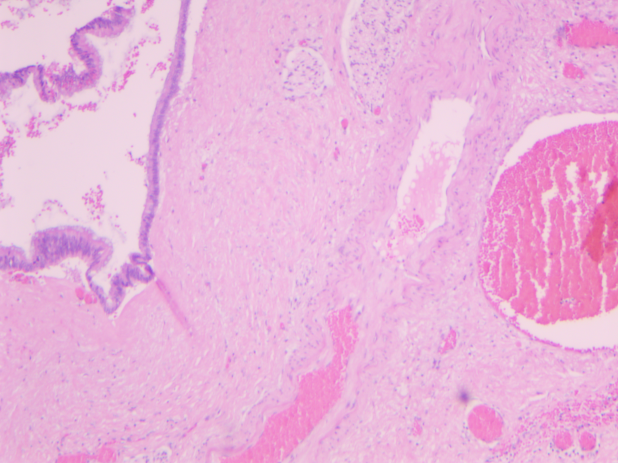 | 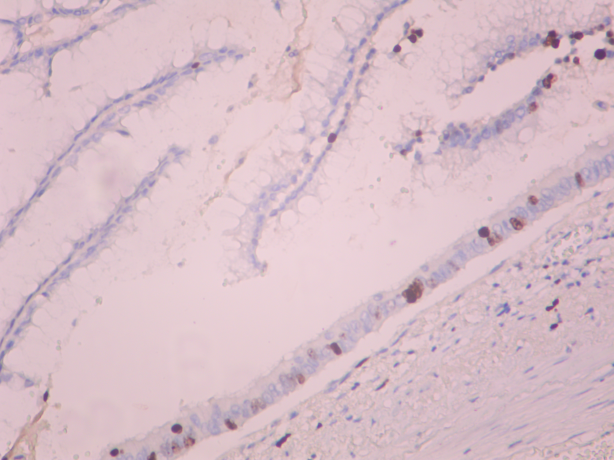 |
| --- | --- |
| H&E staining | KI-67: 8-10% |
| 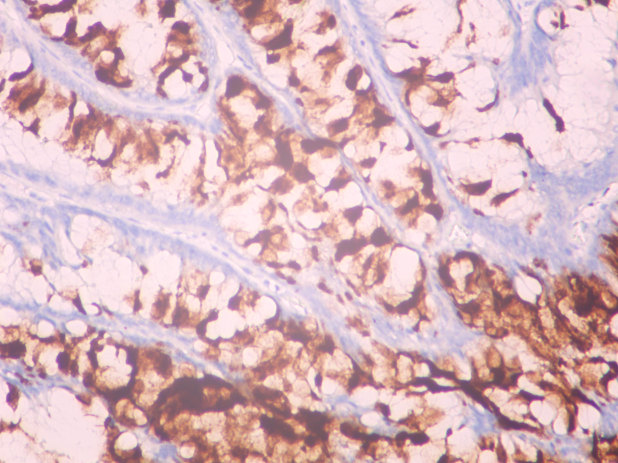 | 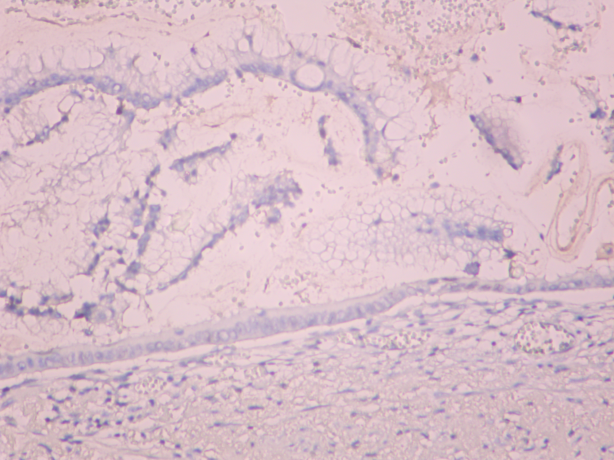 |
| MUC5AC: positive | P53: wild-type expression |

**Figure S3. Histopathological findings from the initial endoscopic biopsy**

| 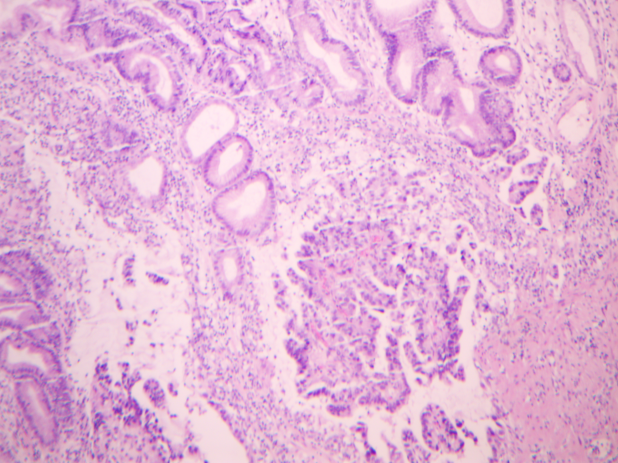 | 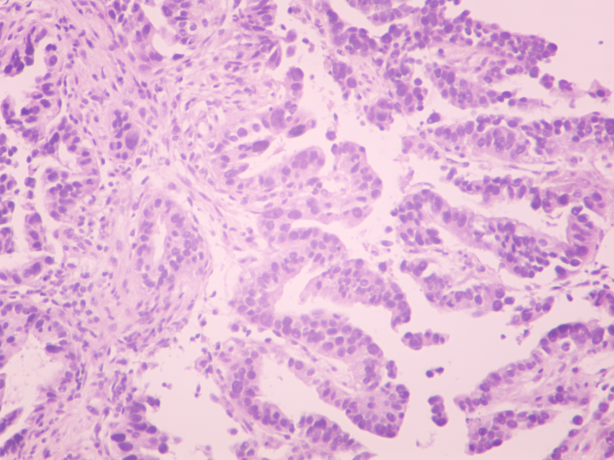 |
| --- | --- |
| H&E staining | H&E staining |
